# Electron streams in air during magnetic-resonance image-guided radiation therapy

**DOI:** 10.1371/journal.pone.0216965

**Published:** 2019-05-15

**Authors:** Hyun Joon An, Jung-in Kim, Jong Min Park

**Affiliations:** 1 Department of Radiation Oncology, Seoul National University Hospital, Seoul, Republic of Korea; 2 Department of Biomedical Sciences, Seoul National University, Seoul, Republic of Korea; 3 Biomedical Research Institute, Seoul National University Hospital, Seoul, Republic of Korea; 4 Institute of Radiation Medicine, Seoul National University Medical Research Center, Seoul, Republic of Korea; 5 Institute for Smart System, Robotics Research Laboratory for Extreme Environments, Advanced Institutes of Convergence Technology, Suwon, Republic of Korea; Northwestern University Feinberg School of Medicine, UNITED STATES

## Abstract

To investigate the undesired irradiations outside of the treatment field by electron streams in air (air-electron-stream) during magnetic-resonance image-guided radiation therapy (MR-IGRT). A custom-made support phantom adjusting angles between the beam central axis (CAX) and the phantom surface (termed phantom-angles), were used. Using the ViewRay system, a rectangular parallelepiped phantom placed on the support phantom, was irradiated with field sizes of 6.3 cm × 6.3 cm (FS6.3) and 12.6 cm × 12.6 cm (FS12.6) at gantry angles of 0°, 30°, and 330°, and phantom-angles of 10°, 20°, and 30°. For each beam delivery, the isocenter was located at the center of mass of the phantom and 3 Gy was delivered to the isocenter (prescription dose = 3 Gy). The doses given by the air-electron-streams were measured using the EBT3 films on the panels placed orthogonal to the direction of the magnetic field at distances of 10 and 17 cm from CAX. Two dose distributions per irradiation were measured on the panel facing the phantom surface of the incident beam (front panel) and on the panel facing the phantom surface of the beam exit (end panel). We investigated the doses by the air-electron-streams by calculating the average doses inside the circles drawn around a point of the maximum dose with radii of x cm (D_Rx_) from the dose distributions on the panels (x = 1–5 cm). The largest value of D_Rx_ was D_R1_ (1.64 Gy, 55% of the prescription dose) at 10 cm distance from CAX, with FS12.6, at 30° phantom-angle and 330° gantry angle. The average difference of the D_R1_ at the end panels (FS12.6) between the calculations and measurements was 1.36 Gy. The average global gamma passing rate with 3%/3 mm on the dose distributions at the end panels (FS12.6) was 40.3%. The calculated dose distributions on both panels were not coincident with the measured dose distributions. The Spearman’s rank correlation coefficients between the projected areas and the D_Rx_ values were always higher than 0.75 (all with *p < 0*.*001)*. The doses by the air-electron-streams increased with the projected areas of the cross-sections of the treatment beams on the panels.

## Introduction

Magnetic-resonance image-guided radiation therapy (MR-IGRT) became clinically available with the release of the first commercial MR-IGRT device, the ViewRay system (ViewRay Inc., Cleveland, OH), which is a combination of an on-board 0.35-T MR imaging system and a radiation therapy system with Co-60 sources [1]. Since this system does not require any extra imaging dose, the daily 3D MRI for patient setup can be acquired [2]. This enables daily adaptive radiation therapy (ART) combined with the rapid optimization algorithm and the rapid dose calculation capability of the ViewRay system [3]. Moreover, near-real-time cine planar MRI can be acquired during treatment with the ViewRay system (a single cine image at 4 frames/s or three cine images at 2 frames/s). Therefore, respiratory gated radiation therapy based on the actual near-real-time tumor motion can be performed without any external surrogate [3]. The ART capability, as well as the gating capability based on the actual tumor motion of the ViewRay system has the potential to minimize the target margins, which is beneficial for sparing doses to a nearby normal tissue around the target volume [4, 5]. This potentially results in reduction in the complications induced by radiation therapy. Furthermore, reduction in the doses given for organs at risk (OARs) by the margin reduction capability of the ViewRay system has the potential to escalate the prescription doses to the target volumes, which potentially increases the efficacy of radiation therapy.

The treatment planning system (TPS) of the ViewRay system is the MRIdian system of which dose calculation algorithm is based on Monte Carlo simulation [6]. Since the Monte Carlo dose calculation algorithm generally calculates doses at the voxels in the region of interest (ROI), *i*.*e*., voxel phantoms, the MRIdian system calculates dose distributions in the ROI, including a patient body as well as air around the patient body. This is a difference of the MRIdian system from other commercial TPSs, which generally calculate dose distributions only inside the body structure. Another feature of the MRIdian system is that it is possible to calculate dose distributions with or without magnetic field (0.35 T magnetic field) [7]. When we generated treatment plans for accelerated partial breast irradiation (APBI) with the magnetic field, we observed low dose streams in air in the direction or opposite direction of the magnetic field (termed air-electron stream) [7]. When we calculated dose distributions without the magnetic field, the directional nature of the low dose stream in air disappeared. Therefore, we concluded that it was generated by the secondary electrons scattered in air, *i*.*e*., charged particles. This phenomenon was observed frequently at the treatment plans for APBI since the target volumes of APBI were generally located close to the surface (sometimes including the patient surface owing to its margin) [7]. Because the energies of the secondary electrons of the ViewRay system are small (the gamma ray energies of Co-60 are 1.17 and 1.33 MeV), when the target volume is located deep in the patient body, the secondary electrons are absorbed in the patient body. However, if the target volume is located close to the surface or includes the patient surface (as in the situation of APBI treatment), the secondary electrons escape the patient body and scatter in air. In this situation, if there is a magnetic field, the scattered secondary electrons in air exactly orthogonal to the direction of magnetic field rotate on the direction of magnetic field due to Lorentz force [8, 9]. If the scattering directions of the secondary electrons have vectors along the magnetic field, the secondary electrons would show helical movements in the direction (or opposite direction) of the magnetic field [10]. Because the energies of the secondary electrons are small, less than 1.33 MeV, the radii of the helix would be small, and therefore, a bunch of these secondary electrons would form the air-electron stream. According to the direction of the vectors of the secondary electrons along the magnetic field, the air-electron stream can be formed in the direction, or opposite direction, of magnetic field. When the air-electron stream was formed in the direction of magnetic field of the ViewRay system (from the couch to the bore) during APBI with a patient position of the head-first supine, the air-electron stream sometimes could reach the patient’s jaw, neck, armpit, or arm [7]. This results in undesired normal tissue irradiation outside of the treatment field.

In this study, we calculated and measured the doses outside of the treatment field by the air-electron streams under various conditions by utilizing a custom-made phantom. We investigated the doses by the air-electron streams as well as the particular conditions to enhance doses by the air-electron streams.

## Materials and methods

### Phantoms and experimental setup

**Fig 1:**
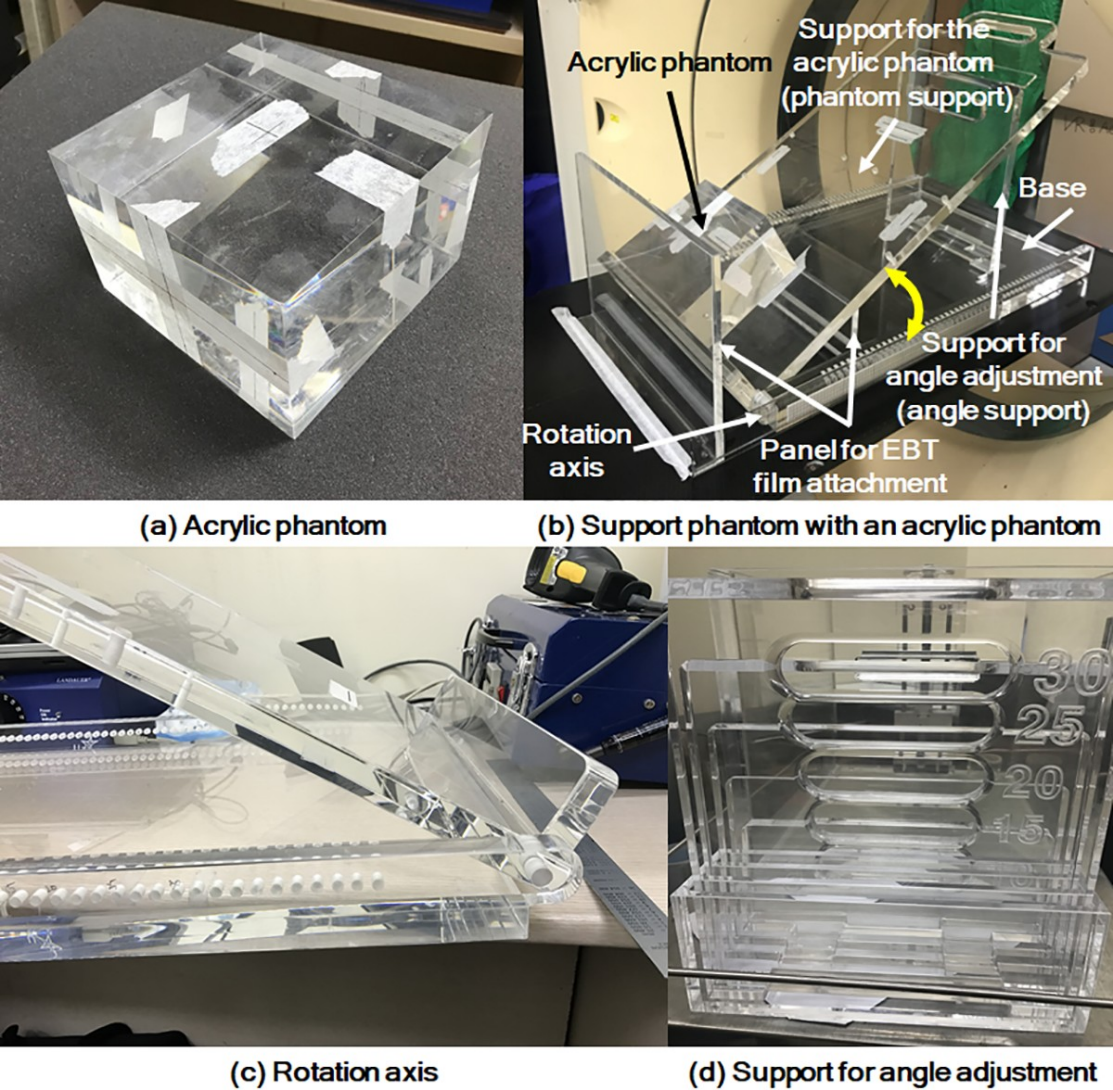
Custom-made acrylic and support phantom.

To investigate doses by the air-electron stream in the magnetic field, we used a custom-made acrylic phantom with dimensions of 15 cm × 15 cm × 10 cm (density of 1.18 g/cm^3^), as shown in Fig 1(A).

**Fig 2:**
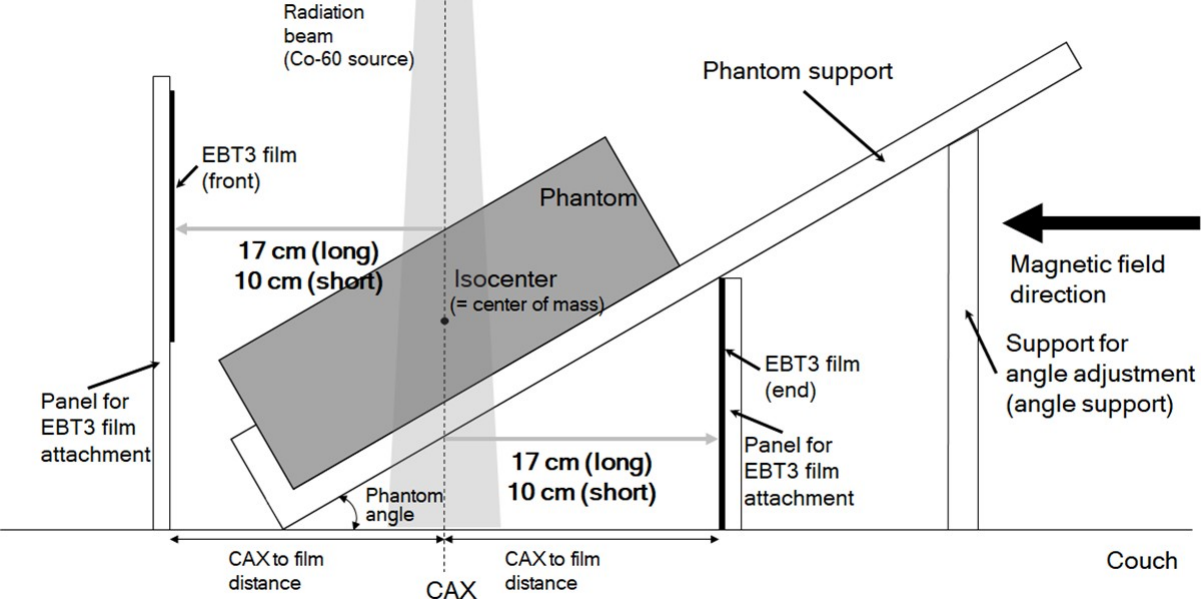
Experimental setup with the phantom and the support phantom.

To vary the angles between the incident beam and the acrylic phantom surface, we designed and fabricated a support phantom as shown in Fig 1(B) and Fig 2. Whole parts of the support phantom were made of acrylic to be compatible with the magnetic field. The support for the acrylic phantom (termed *phantom support*) was laid over the base of the support phantom, and they were connected to each other by a rotation axis, as shown in Fig 1(C). We cut grooves at the base to fix the supports for angle adjustments, termed *angle support* (Fig 1(D)), to the base. The *angle support* can be put into the corresponding groove at the base to form a particular angle between the *phantom support* and the base. By combination of the angle support and its corresponding groove, angles ranging from 5° to 30° at intervals of 5° can be generated between the *phantom support* and the base. This means that the support phantom can adjust the angles between the couch surface and the surface of the acrylic phantom placed on the *phantom support* from 5° to 30° at 5° intervals since the acrylic phantom surface is parallel to the surface of the *phantom support*. Since the angle between the central axis (CAX) and the vector orthogonal to the acrylic phantom surface (termed *phantom angle*) is same as that between the phantom surface and the couch surface, the *phantom angles* can be adjusted from 5° to 30° at 5° intervals. To measure doses by the air-electron stream, two panels for the attachment of Gafchromic EBT3 films (Ashland ISP Advanced Materials, Wayne, NJ) were setup vertically to the base, as shown in Fig 1(B). Experimental setup with the phantom and the support phantom is shown in Fig 2.

The panels for the attachment of EBT3 films were located parallel to CAX and vertical to the direction of magnetic field. We set the panels to be parallel to each other and to be located at the same distances from CAX, which were 17 cm (long-distance setup) and 10 cm (short-distance setup). The panel in front of the incident beam cross-section was termed *front panel* and that facing the exit beam cross-section was termed *end panel*.

### Conditions of beam irradiation

All beam irradiations in the present study were performed with the ViewRay system. For every beam irradiation, the isocenter located at 105 cm distance from the radiation source (source to axis distance, SAD = 105 cm) was always located at the center of mass of the acrylic phantom [1]. To investigate the effect of the *phantom angles* on the doses by the air-electron stream, the *phantom angles* tested in this study were 10°, 20°, and 30° (three *phantom angles*). At each phantom angle, Co-60 beams with square field sizes of 6.3 cm × 6.3 cm and 12.6 cm × 12.6 cm were delivered to the acrylic phantom (two field sizes) to investigate the field size effect on the doses by the air-electron stream. For each field size, gantry angles of 0°, 30°, and 330° (three gantry angles) were chosen. For each beam delivery, doses delivered to the surfaces of the *front panel* and *end panel* were measured with EBT3 films (two measurements for a single beam delivery). The surface doses of the *front panel* and *end panel* were measured at distances of 10 and 17 cm from CAX (two distances) under identical beam delivery condition. Therefore, 36 beams were delivered to the acrylic phantom (3 *phantom angles* × 2 field sizes × 3 gantry angles × 2 distances from CAX) and 72 dose distributions (× *front* and *end panels*) were measured. For the short-distance setup (panel setup at 10 cm distance from CAX) while adjusting the *phantom angles* from 10° to 30°, we chose the acrylic phantom (15 cm × 15 cm × 10 cm) for this study, of which dimension was smaller than those of the commercial solid water phantoms (30 cm × 30 cm × various thicknesses). For each irradiation in this study, 3 Gy was delivered to the isocenter located at the center of mass of the acrylic phantom; that is, the prescription dose was 3 Gy.

### Dose calculations

We acquired CT image sets of the phantom with the support phantom to calculate dose distributions at the surfaces of the *front* and *end panels*. Since there were six experimental geometries in the present study (3 *phantom angles* × 2 distances between the panels and CAX), we acquired six CT image sets of the phantom with the support phantom by using the Brilliance CT Big Bore (Phillips, Amsterdam, Netherlands) with a slice thickness of 1 mm. With the CT images, dose distributions were calculated with the MRIdian system under the identical conditions as the measurements. Treatment plans were generated to deliver 3 Gy to the isocenter under each beam delivery condition. Dose distributions were calculated with a dose calculation grid size of 3 mm, which is recommended by the manufacturer for an optimal dose calculation [4, 6]. To maintain the dose calculation accuracy with the Monte Carlo dose calculation algorithm of the MRIdian system while maintaining the dose calculation speeds to be appropriate for on-table ART, the ViewRay Inc. recommends to use a dose calculation grid size of 3 mm in the clinical setting.

### Gafchromic EBT3 film measurements

The dose response of the EBT3 films was calibrated with the ViewRay system under the magnetic field to eliminate the magnetic field effect on the EBT3 films [11]. The dual channel method for red and blue corrections was applied (spatial resolution = 75 dpi) [12]. According to the previous studies, uniformity correction for the scanner was applied [13, 14]. A flatbed scanner, Epson 10000XL scanner (Epson Canada Ltd., Toronto, Ontario, Canada), was used for scanning the EBT3 films. The films were scanned in 48-bit color mode, *i*.*e*., RGB mode, after 20 h of irradiation. The scanned dose distributions were analyzed with the RIT 113 software (Radiological Imaging Technology, Inc., Colorado Springs, CO).

### Data analysis

At both the measured and calculated dose distributions on the surfaces of the *front* and *end panels*, the points of the maximum doses were found. After that, circles were drawn around the point of the maximum dose with radii extending from 1 to 4 cm with an interval of 1 cm. For each circle, average doses inside the circles were calculated. Therefore, the circle with a radius of 1 cm was the highest dose region in the dose distributions because there was a single inflection point in the dose distributions of the present study. The average dose inside the circle with a radius of *x* cm was termed D_Rx_. Naturally, the value of D_R1_ was the highest and that of D_R4_ was the lowest. In addition, we calculated areas of isodose lines of 30% (0.9 Gy), 50% (1.5 Gy), 70% (2.1 Gy), 90% (2.7 Gy), and 100% of the prescription dose (3 Gy) in the dose distributions. The area of an isodose line of *y*% of 3 Gy in cm^2^ was termed A_y%_.

To examine the differences between the calculated and measured doses, we performed global gamma evaluation with absolute doses. A gamma criterion of 3%/3 mm was used and points with doses equal to or less than 10% of the maximum dose in the dose distribution were not evaluated, *i*.*e*., the threshold dose was 10%. In addition, we calculated percent differences in the values of D_Rx_ between the calculated and measured dose distributions. For A_y%_, we acquired just the differences by subtracting the A_y%_ value of the measured dose from that of the calculated dose.

To investigate the tendency of doses by the air-electron stream, we acquired percent differences in D_Rx_ as well as in the A_y%_ between the *front* and *end panels*, between the large and small field sizes (6.3 cm × 6.3 cm vs. 12.6 cm × 12.6 cm), and between the long and short distances from CAX (10 cm vs. 17 cm).

To analyze doses by the air-electron streams combined with the projected areas of the cross-sections of the treatment beams at the phantom surface on the panels, we mathematically calculated the projected areas on both the *front* and *end* panels under various conditions of the present study. For the *end panels*, the cross-sections of the exit beam at the support phantom beneath the acrylic phantom were calculated since the acrylic phantom was placed on the support phantom and the beam cross-section at the support phantom was where the secondary electrons were scattered in air. After that, correlations between the doses by the air-electron streams and the projected areas on the panels were analyzed by calculating Spearman’s rank correlation coefficients (*r*) with the corresponding *p* values.

## Results

### Doses on panel surfaces (irradiation outside of the treatment field)

#### End panel surface dose with a field size of 6.3 cm × 6.3 cm

**Fig 3:**
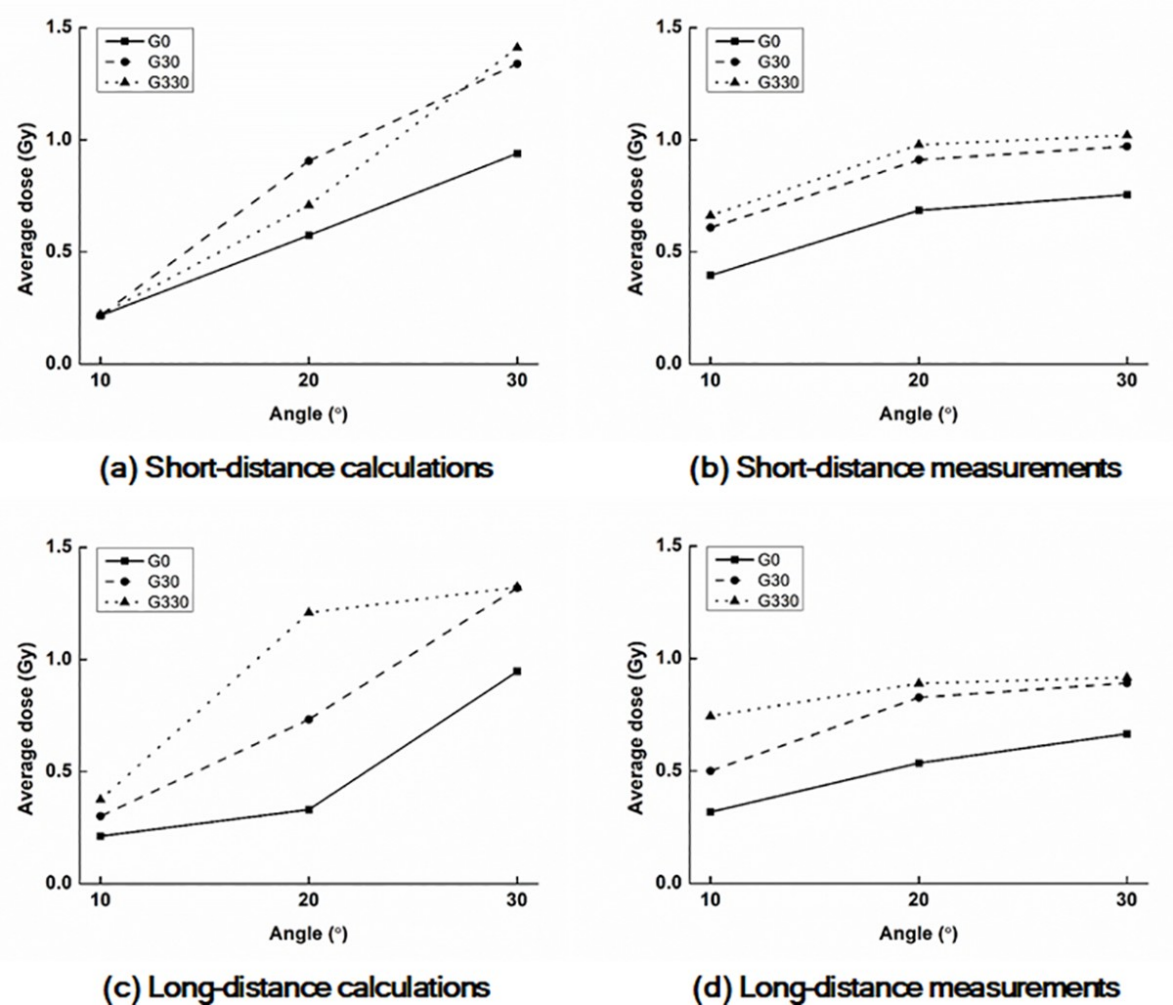
Plots of average doses of D_R1_ with FS6.3 on the surfaces of the end panel. Calculated values at the 10 cm (a) and 17 cm (c) distances from the central axis as well as measured values at the 10 cm (b) and 17 cm (d) distances. G0, G30, and G330 indicate gantry angles of 0°, 30°, and 330°, respectively.

The values of D_Rx_ and A_y%_ with a field size of 6.3 cm × 6.3 cm acquired from the calculated and measured dose distributions on the surfaces of the *end panels* are shown in Tables 1 and 2, respectively. D_R1_ with a field size of 6.3 cm × 6.3 cm acquired from the calculated and measured dose distributions on the surfaces of the *end panels* are plotted according to the *phantom angles* shown in Fig 3.

**Table 1:** Average doses in the circles drawn around the point of the maximum dose at the end panels with a field size of 6.3 cm × 6.3 cm.

		Calculated values (cGy)				Measured values (cGy)			
Phantom angle (°)	Gantry angle (°)	D_R1_	D_R2_	D_R3_	D_R4_	D_R1_	D_R2_	D_R3_	D_R4_
17 cm from the CAX									
10	30	30.1	22.1	17.7	15.5	50.0	32.5	25.1	21.0
	0	21.2	16.5	13.9	12.5	31.8	21.0	16.9	14.8
	330	37.5	26.8	21.3	18.8	74.4	51.8	41.5	36.4
20	30	73.3	51.8	35.9	26.3	82.7	65.1	47.0	33.8
	0	33.1	26.3	20.4	16.2	53.5	41.1	29.9	21.9
	330	120.9	92.1	67.5	50.4	89.0	70.6	51.2	38.0
30	30	131.9	117.1	94.8	71.8	89.1	81.1	65.2	48.4
	0	94.7	85.7	70.8	55.5	66.6	55.9	44.0	33.7
	330	132.2	118.1	96.2	74.0	91.6	83.3	67.8	52.3
10 cm from the CAX									
10	30	21.7	20.8	20.2	19.7	60.8	50.8	46.9	42.3
	0	21.6	20.7	20.0	19.5	39.5	35.6	33.9	31.7
	330	22.0	21.1	20.7	20.3	66.3	56.5	52.8	49.3
20	30	90.6	64.3	50.2	43.0	91.1	76.9	63.0	53.7
	0	57.4	38.6	30.3	27.3	68.6	54.2	45.0	40.4
	330	70.9	58.2	49.3	45.3	97.8	82.0	67.7	59.8
30	30	133.9	117.5	95.5	76.9	97.1	86.1	71.8	59.0
	0	94.0	84.6	72.1	61.1	75.5	66.6	55.8	47.7
	330	141.1	113.6	89.2	71.8	102.0	90.9	78.0	65.3

*Abbreviations*: D_Rx_ = average dose in a circle drawn around the point of the maximum dose with a radius of *x* cm, CAX = central axis

**Table 2:** Areas of isodose lines of *x*% of the prescription dose (3 Gy) calculated from both the calculated and measured dose distributions at the end panels with a field size of 6.3 cm × 6.3 cm.

		Calculated values (cm^2^)	Measured values (cm^2^)
Phantom angle (°)	Gantry angle (°)	A_30%_	A_30%_
17 cm from CAX			
20	30	0.2	-
	330	9.6	3.1
30	30	17.0	1.4
	0	6.0	-
	330	18.9	3.6
10 cm from CAX			
20	30	2.7	3.6
	330	-	6.5
30	30	16.5	6.9
	0	5.5	-
	330	12.0	9.2

*Abbreviations*: A_y%_ = area of an isodose line of *y*% of the prescription dose, CAX = central axis

**Fig 4:**
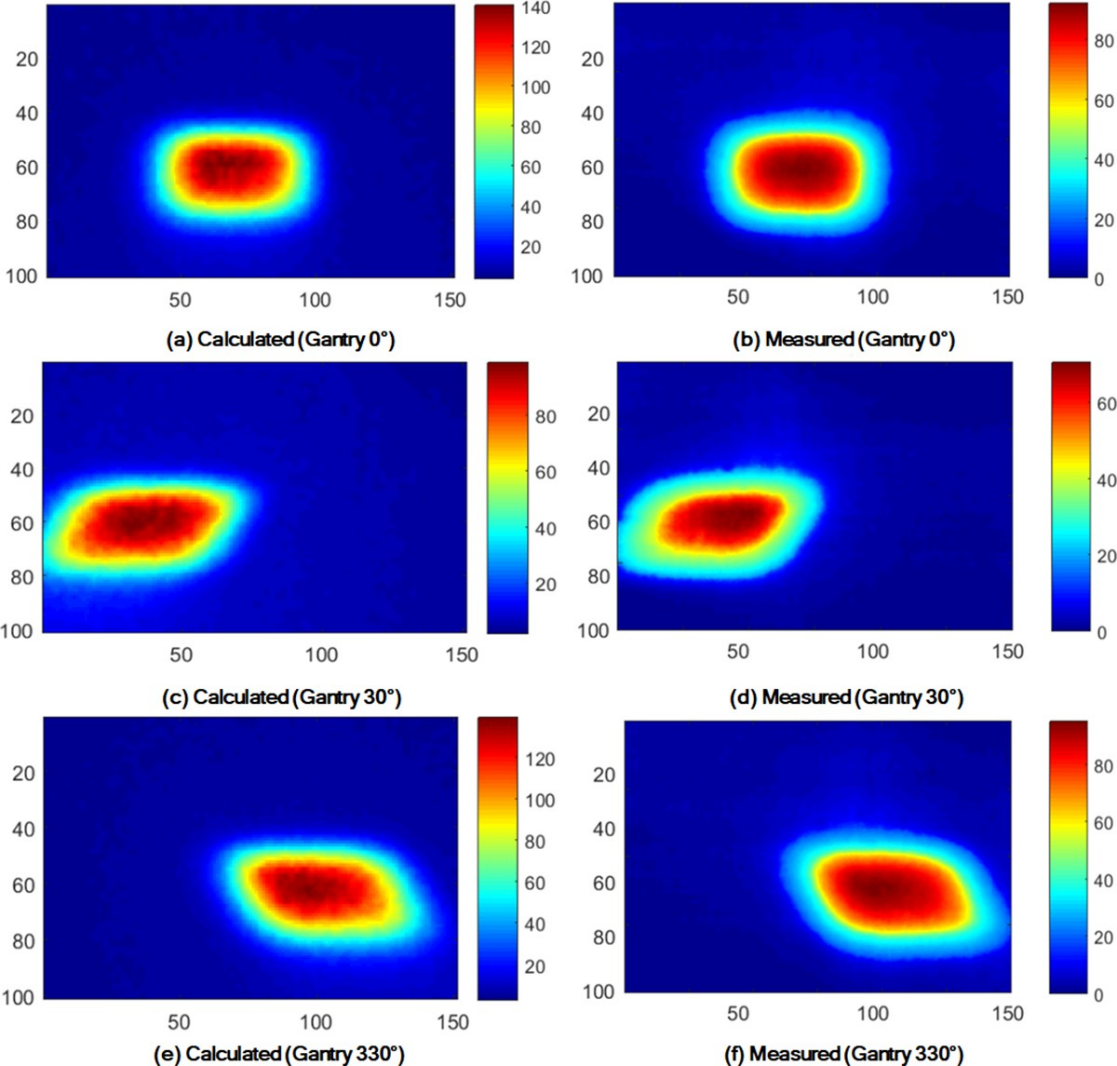
Dose distributions of the air-electron stream calculated in TPS and measured with EBT3 film. The dose distributions were acquired on the end panel with a field size of 6.3 cm × 6.3 cm, at a phantom angle of 30°, and at 17 cm distance from CAX. The unit of each legend is cGy and the numbers of x and y axes are pixel numbers (pixel size = 1.36 mm × 1.36 mm).

An example of dose distributions calculated in TPS, as well as measured with the EBT3 films, is shown in Fig 4.

According to its definition, the values of D_Rx_ decreased with increasing radii of the circles and the values of A_y%_ decreased with increasing value of *y*.

The values of D_Rx_ as well as A_y%_ with a gantry angle of 0° were always significantly smaller than those with gantry angles of 30° and 330°. There were huge discrepancies in the values of both D_Rx_ and A_y%_ between the calculated and measured dose distributions. With increasing *phantom angles*, the values of D_Rx_ also increased. For the calculated and measured doses, the maximum D_Rx_ values were the values of D_R1_ with a *phantom angle* of 30°, gantry angle of 330°, and 10 cm distance from CAX, which were 1.41 Gy (47% of the prescription dose) and 1.02 Gy (34% of the prescription dose), respectively.

In the case of A_y%_, isodose lines equal to or larger than 50% of the prescription dose were not observed for both the calculation and measurements. The values of A_y%_ with a gantry angle of 0° were always lower than those with gantry angles of 30° and 330°. The maximum value of A_y%_ from the measured dose distributions was A_30%_ with a *phantom angle* of 30°, gantry angle of 330°, and 10 cm distance from CAX, which was 9.2 cm^2^.

#### End panel surface dose with a field size of 12.6 cm × 12.6 cm

**Fig 5:**
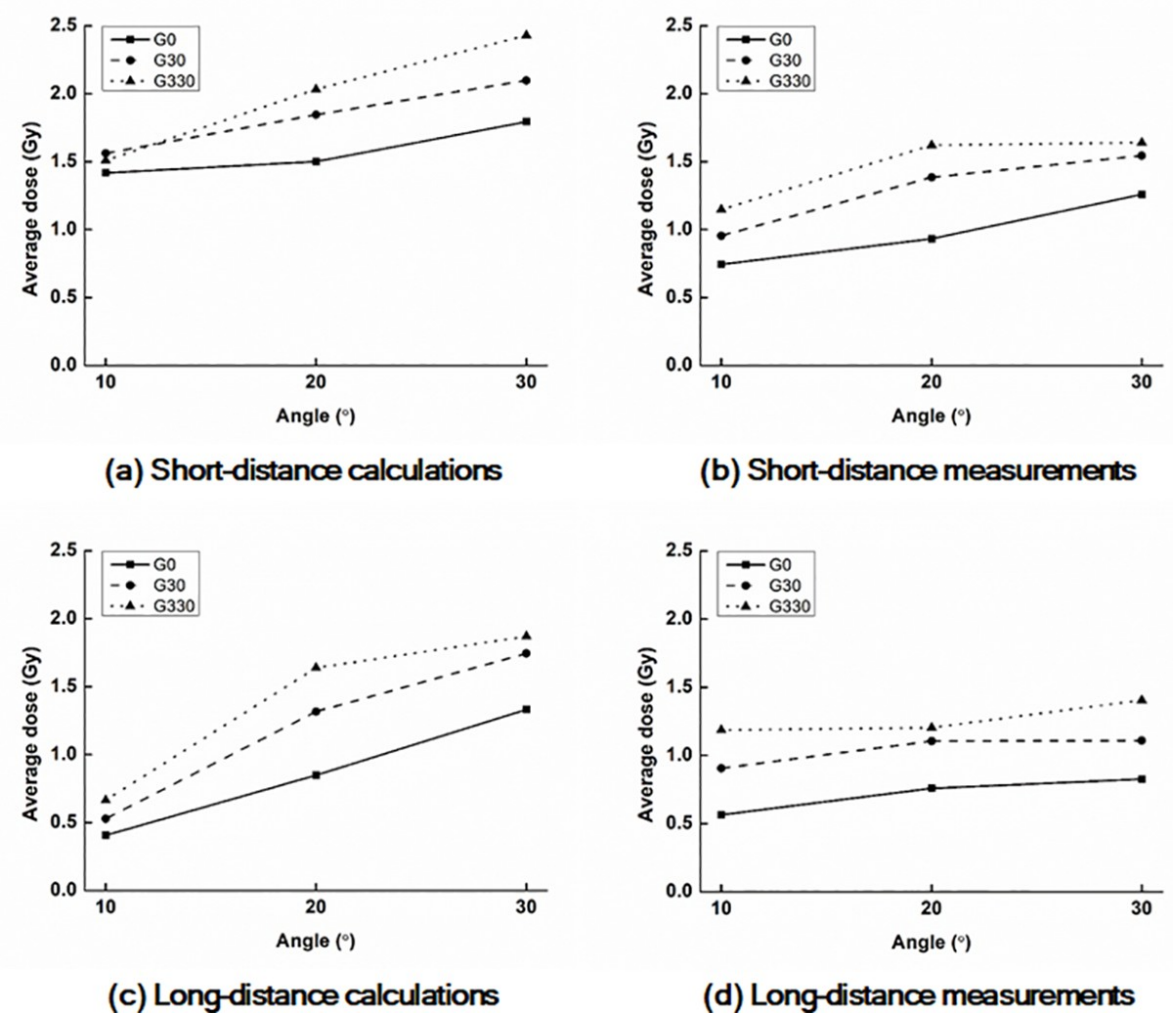
Plots of average doses of D_R1_ with FS12.6 on the surfaces of the end panel. Calculated values at the 10 cm (a) and 17 cm (c) distances from CAX as well as measured values at the 10 cm (b) and 17 cm (d) distances. G0, G30, and G330 indicate gantry angles of 0°, 30°, and 330°, respectively.

The values of D_Rx_ and A_y%_ with a field size of 12.6 cm × 12.6 cm acquired from the calculated and measured dose distributions on the surfaces of the *end panels* are shown in Tables 3 and 4, respectively. D_R1_ with a field size of 12.6 cm × 12.6 cm acquired from the calculated and measured dose distributions on the surfaces of the *end panels* are plotted according to the *phantom angles* in Fig 5.

**Table 3:** Average doses in the circles drawn around the point of the maximum dose at the end panels with a field size of 12.6 cm × 12.6 cm.

		Calculated values (cGy)				Measured values (cGy)			
Phantom angle (°)	Gantry angle (°)	D_R1_	D_R2_	D_R3_	D_R4_	D_R1_	D_R2_	D_R3_	D_R4_
17 cm from the CAX									
10	30	52.8	45.2	39.9	38.3	90.6	72.5	62.2	58.4
	0	40.7	36.4	33.0	32.0	56.4	43.4	36.8	34.2
	330	66.4	54.0	46.4	44.1	118.7	94.6	80.4	75.2
20	30	131.7	107.9	90.6	78.1	110.6	100.0	87.6	76.4
	0	84.8	69.2	57.5	49.1	75.9	70.8	62.5	53.9
	330	163.9	139.1	116.0	99.0	120.4	111.2	99.8	82.4
30	30	174.5	159.5	139.4	121.0	111.0	102.3	93.0	84.4
	0	133.2	112.9	98.3	89.5	82.7	77.4	68.1	58.3
	330	187.0	165.8	139.1	115.8	140.6	122.7	102.0	89.9
10 cm from the CAX									
10	30	156.2	148.2	146.5	144.5	95.5	91.7	90.6	88.5
	0	141.9	136.0	134.9	133.5	74.5	68.3	63.4	59.6
	330	150.9	144.3	143.1	142.2	114.9	107.7	104.2	99.9
20	30	184.6	154.8	147.0	145.0	138.6	124.1	117.6	114.8
	0	150.1	139.8	135.0	134.0	93.3	84.3	76.0	70.1
	330	203.3	173.1	149.3	144.0	162.2	136.1	119.0	109.1
30	30	209.8	192.5	181.7	179.7	154.5	140.0	127.4	121.1
	0	179.5	161.7	143.5	136.0	126.0	105.8	91.8	82.5
	330	243.0	220.7	190.1	161.9	164.1	144.4	119.8	110.0

*Abbreviations*: D_Rx_ = average dose in a circle drawn around the point of the maximum dose with a radius of *x* cm, CAX = central axis

**Table 4:** Areas of isodose lines of *x*% of the prescription dose (3 Gy) calculated from both the calculated and measured dose distributions at the end panels with a field size of 12.6 cm × 12.6 cm.

		Calculated values (cm^2^)			Measured values (cm^2^)		
Phantom angle (°)	Gantry angle (°)	A_70%_	A_50%_	A_30%_	A_70%_	A_50%_	A_30%_
17 cm from the CAX							
10	30	-	-	-	-	-	5.2
	330	-	-	-	-	-	23.6
20	30	-	-	20.6	-	-	31.6
	0	-	-	1.7	-	-	-
	330	-	15.4	48.1	-	-	51.6
30	30	-	31.3	95.9	-	-	42.4
	0	-	0.4	76.2	-	-	-
	330	-	35.1	105.3	-	-	57.2
10 cm from CAX							
10	30	-	8.9	28.6	-	-	10.5
	0	-	1.0	27.3	-	-	-
	330	-	8.9	34.0	-	-	21.2
20	30	-	15.7	42.7	-	-	39.0
	0	-	4.3	49.3	-	-	11.9
	330	1.6	13.2	42.5	-	17.1	51.7
30	30	12.7	55.7	85.9	-	9.6	60.0
	0	-	43.7	86.5	-	-	45.5
	330	26.0	48.4	99.2	-	18.5	73.7

*Abbreviations*: A_y%_ = area of an isodose line of *y*% of the prescription dose, CAX = central axis

For the doses with a field size of 12.6 cm × 12.6 cm, the same tendency as that with a field size of 6.3 cm × 6.3 cm was observed, but the absolute values of D_Rx_ and A_y%_ with a field size of 12.6 cm × 12.6 cm were always higher than those with a field size of 6.3 cm × 6.3 cm. The maximum D_Rx_ value was D_R1_ with a *phantom angle* of 30°, gantry angle of 330°, and the 10 cm distance (2.43 Gy and 1.64 Gy for the calculated and measured values, respectively). The maximum value of A_y%_ from the calculated dose distributions was A_70%_ (26 cm^2^) at the *phantom angle* of 30°, gantry angle of 330°, and 10 cm distance from CAX; however, the values of A_70%_ from the measured dose distributions were always zero. From the measurements, A_50%_ at a *phantom angle* of 30°, gantry angle of 330°, and 10 cm distance from CAX was the largest (18.5 cm^2^).

#### Front panel surface dose with a field size of 6.3 cm × 6.3 cm

**Fig 6:**
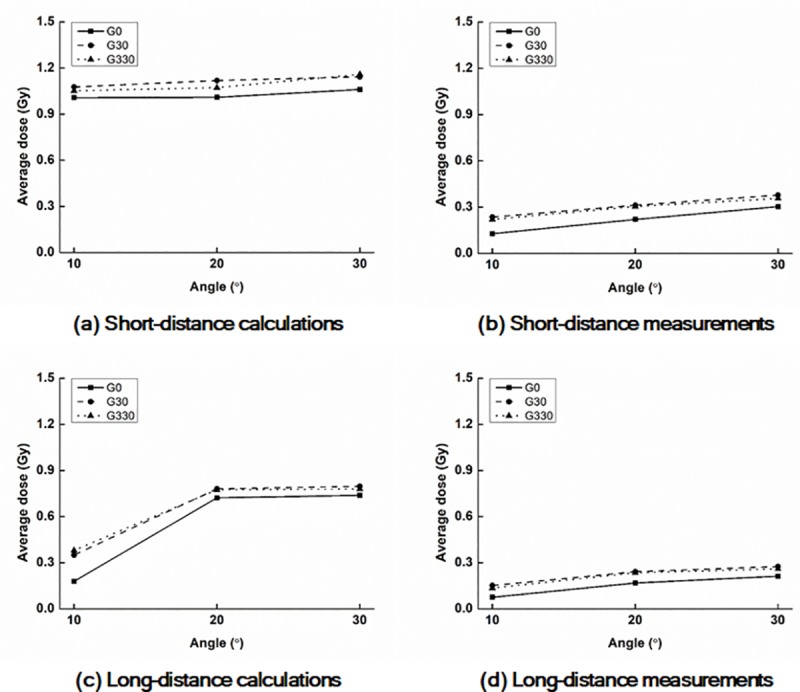
Plots of average doses of D_R1_ with FS 6.3 on the surfaces of the front panel. Calculated values at the 10 cm (a) and 17 cm (c) distances from CAX as well as measured values at the 10 cm (b) and 17 cm (d) distances. G0, G30, and G330 indicate gantry angles of 0°, 30°, and 330°, respectively.

The values of D_Rx_ and A_y%_ with a field size of 6.3 cm × 6.3 cm acquired from the calculated and measured dose distributions on the surfaces of the *front panels* are shown in Tables 5 and 6, respectively. The D_R1_ with a field size of 6.3 cm × 6.3 cm acquired from the calculated and measured dose distributions on the surfaces of the *front panels* are plotted according to the *phantom angles* in Fig 6.

**Table 5:** Average doses in the circles drawn around the point of maximum dose at the front panels with a field size of 6.3 cm × 6.3 cm.

		Calculated values (cGy)				Measured values (cGy)			
Phantom angle (°)	Gantry angle (°)	D_R1_	D_R2_	D_R3_	D_R4_	D_R1_	D_R2_	D_R3_	D_R4_
17 cm from the CAX									
10	30	35.0	26.3	21.1	18.0	15.2	10.2	7.8	6.6
	0	17.9	13.4	10.0	7.5	7.5	5.9	4.5	3.3
	330	38.0	29.2	25.4	19.7	13.5	8.8	6.7	5.2
20	30	78.1	75.3	71.0	61.4	24.1	19.4	15.3	12.9
	0	72.3	69.4	63.0	54.9	16.8	13.2	10.5	8.1
	330	77.5	71.9	68.5	59.5	23.5	21.4	17.7	13.9
30	30	79.7	77.6	71.3	66.5	27.5	25.3	20.4	15.9
	0	73.9	70.7	64.0	55.8	21.2	17.3	14.0	10.8
	330	77.9	74.8	71.2	64.3	26.0	23.1	18.9	15.0
10 cm from CAX									
10	30	107.7	99.8	87.8	75.8	23.5	19.2	17.2	15.7
	0	100.7	88.7	72.7	61.3	12.8	9.3	7.9	6.9
	330	105.2	96.0	83.0	70.2	22.0	18.0	16.5	14.9
20	30	111.9	107.8	99.1	86.4	31.2	27.8	23.4	20.6
	0	101.0	94.6	82.3	63.8	22.0	20.8	17.3	13.5
	330	107.3	97.0	84.4	72.5	30.4	25.4	22.7	19.5
30	30	114.3	109.6	101.0	88.4	37.8	34.9	30.8	26.4
	0	106.1	99.5	86.1	65.5	30.4	29.0	27.4	25.0
	330	115.9	111.4	100.5	83.3	35.6	32.7	29.0	26.1

*Abbreviations*: D_Rx_ = average dose in a circle drawn around the point of maximum dose with a radius of *x* cm, CAX = central axis

**Table 6:** Areas of isodose lines of *x*% of the prescription dose (3 Gy) calculated from both the calculated and measured dose distributions at the front panels with a field size of 6.3 cm × 6.3 cm.

		Calculated values (cm^2^)	Measured values (cm^2^)
Phantom angle (°)	Gantry angle (°)	A_30%_	A_30%_
10 cm from CAX			
10	30	6.4	-
	0	3.9	-
	330	4.1	-
20	30	16.7	-
	0	6.7	-
	330	12.5	-
30	30	20.2	-
	0	8.3	-
	330	17.6	-

*Abbreviations*: A_y%_ = area of an isodose line of *y*% of the prescription dose, CAX = central axis

For the doses at the *front panel* with a field size of 6.3 cm × 6.3 cm, the same tendency as that for the *end panel* was observed, but the absolute values of D_Rx_ and A_y%_ of the *front panel* were always smaller than those of the *end panel*. The maximum D_Rx_ value from the calculated and measured dose distributions were D_R1_ at the *phantom angle* of 30°, gantry angle of 330°, and 10 cm distance, which were 1.16 Gy (39% of the prescription dose) and 0.36 Gy (12% of the prescription dose). For the calculated dose distributions, the A_30%_ at the *phantom angle* of 30°, gantry angle of 30°, and 10 cm distance was the largest (20.2 cm^2^), but the values of A_30%_ from the measured dose distributions were always zero.

#### Front panel surface dose with a field size of 12.6 cm × 12.6 cm

**Fig 7:**
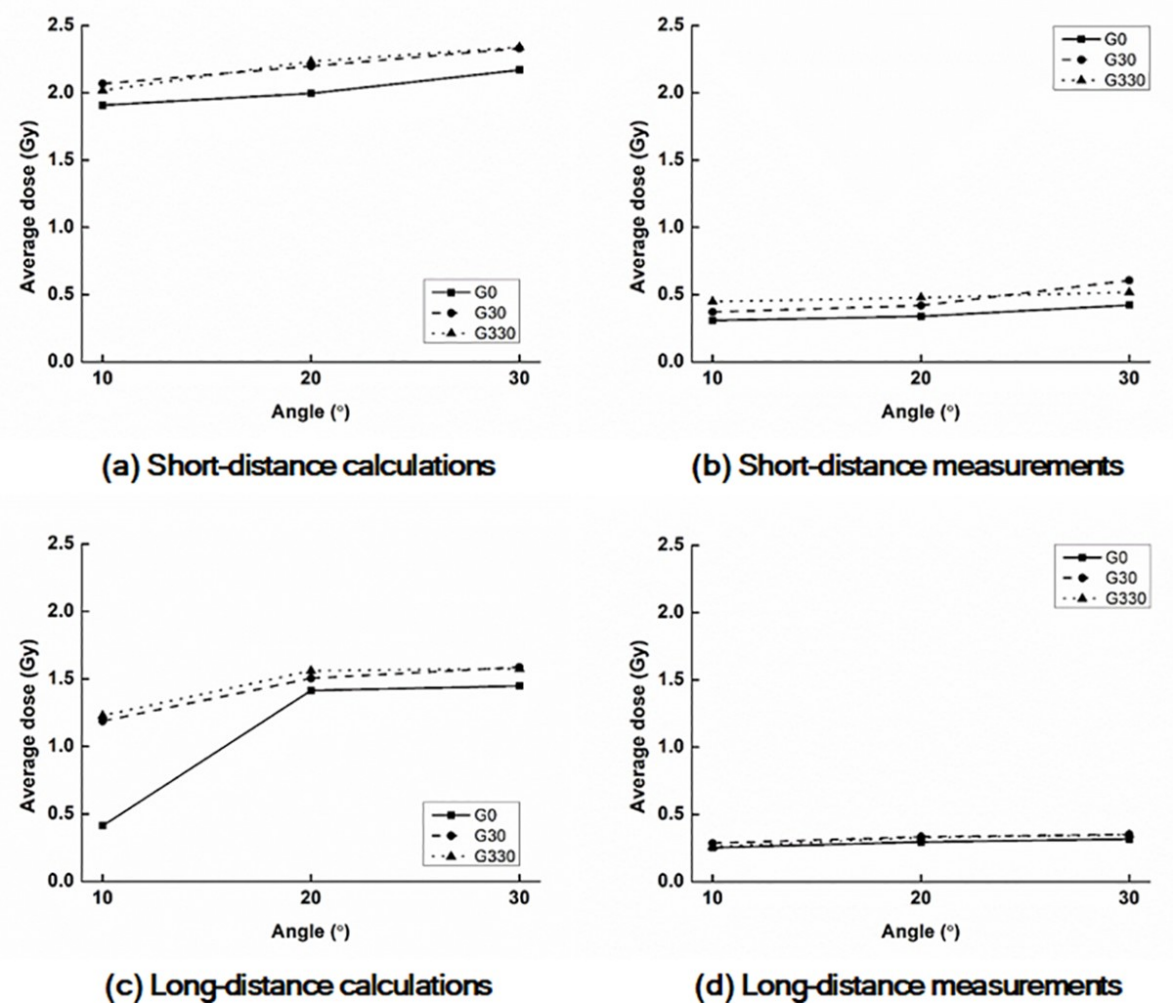
Plots of average doses of D_R1_ with FS12.6 on the surfaces of the front panel. Calculated values at the 10 cm (a) and 17 cm (c) distances from CAX as well as measured values at the 10 cm (b) and 17 cm (d) distances. G0, G30, and G330 indicate gantry angles of 0°, 30°, and 330°, respectively.

The values of D_Rx_ and A_y%_ with a field size of 12.6 cm × 12.6 cm acquired from the calculated and measured dose distributions on the surfaces of the *front panels* are shown in Tables 7 and 8, respectively. The D_R1_ with a field size of 12.6 cm × 12.6 cm acquired from the calculated and measured dose distributions on the surfaces of the *front panels* are plotted according to the *phantom angles* in Fig 7.

**Table 7:** Average doses in the circles drawn around the point of the maximum dose at the front panels with a field size of 12.6 cm × 12.6 cm.

		Calculated values (cGy)				Measured values (cGy)			
Phantom angle (°)	Gantry angle (°)	D_R1_	D_R2_	D_R3_	D_R4_	D_R1_	D_R2_	D_R3_	D_R4_
17 cm from CAX									
10	30	118.8	82.2	59.7	45.0	28.6	27.0	23.4	21.6
	0	41.2	36.5	30.6	25.3	25.3	21.4	16.7	12.9
	330	122.9	84.8	61.3	48.2	26.1	23.8	20.2	18.5
20	30	150.5	149.4	148.2	145.9	33.3	32.1	31.3	30.8
	0	141.5	140.1	137.1	124.5	29.5	28.6	27.8	26.6
	330	156.1	151.9	145.7	141.4	32.8	31.7	29.7	29.0
30	30	158.8	157.8	156.4	154.8	35.1	33.0	31.9	31.0
	0	144.9	142.0	137.6	132.4	31.6	30.5	29.4	27.7
	330	157.7	155.6	153.6	151.4	34.8	32.5	30.0	29.7
10 cm from CAX									
10	30	206.9	192.5	170.5	157.9	37.1	35.3	34.2	32.8
	0	190.8	169.2	147.5	141.6	30.8	28.3	27.0	25.8
	330	201.8	189.0	163.5	143.1	44.9	36.4	33.8	32.8
20	30	219.8	218.4	216.6	212.5	41.9	40.4	39.2	37.9
	0	199.7	194.3	184.1	164.7	33.9	32.8	31.5	29.9
	330	223.3	221.3	210.6	184.5	47.8	38.0	34.8	33.8
30	30	233.1	231.2	229.5	224.9	60.6	57.6	54.9	52.6
	0	217.1	211.2	200.9	177.6	42.3	40.1	38.2	36.6
	330	233.9	228.3	219.0	215.0	51.8	41.4	38.4	37.9

*Abbreviations*: D_Rx_ = average dose in a circle drawn around the point of maximum dose with a radius of *x* cm, CAX = central axis

**Table 8:** Areas of isodose lines of *x*% of the prescription dose (3 Gy) calculated from both the calculated and measured dose distributions at the front panels with a field size of 12.6 cm × 12.6 cm.

		Calculated values (cm^2^)			Measured values (cm^2^)		
Phantom angle (°)	Gantry angle (°)	A_70%_	A_50%_	A_30%_	A_70%_	A_50%_	A_30%_
17 cm from the CAX							
10	30	-	-	5.4	-	-	-
	0	-	-	-	-	-	-
	330	-	-	8.9	-	-	-
20	30	-	18.8	82.0	-	-	-
	0	-	0.3	54.2	-	-	-
	330	-	21.7	77.4	-	-	-
30	30	-	28.1	83.3	-	-	-
	0	-	0.5	94.0	-	-	-
	330	-	23.9	113.3	-	-	-
10 cm from CAX							
10	30	0.7	18.1	25.3	-	-	-
	0	-	11.7	18.7	-	-	-
	330	0.4	15.0	20.2	-	-	-
20	30	26.9	48.0	59.6	-	-	-
	0	-	28.6	38.9	-	-	-
	330	20.3	37.8	48.6	-	-	-
30	30	28.1	60.6	77.5	-	-	-
	0	8.5	29.1	41.0	-	-	-
	330	24.2	54.5	69.4	-	-	-

*Abbreviations*: A_y%_ = area of an isodose line of *y*% of the prescription dose, CAX = central axis

For the doses at the *front panel* with a field size of 12.6 cm × 12.6 cm, the same tendency as those of the other results was observed. The absolute values of D_Rx_ and A_y%_ of the *front panel* with a field size of 12.6 cm × 12.6 cm were always larger than those with a field size of 6.3 cm × 6.3 cm. However, the absolute values of D_Rx_ and A_y%_ of the *front panel* with a field size of 12.6 cm × 12.6 cm were always smaller than those of the *end panel* with the same field size. The maximum D_Rx_ value from the calculated dose distributions was D_R1_ with a *phantom angle* of 30°, gantry angle of 330°, and 10 cm distance, which was 2.34 Gy (78% of the prescription dose). The maximum D_Rx_ value from the measured dose distributions was D_R1_ with a phantom angle of 30°, gantry angle of 30°, and 10 cm distance, which was 0.61 Gy (20% of the prescription dose). For the A_y%_ from the calculated dose distributions, A_70%_ at a *phantom angle* of 30°, gantry angle of 30°, and 10 cm distance was the largest (28.1 cm^2^); however, even A_30%_ was always zero in the case of measurements.

### Differences between the calculation and measurement

#### Results of gamma evaluation

The average global gamma passing rates with 3%/3 mm on the dose distributions at the *end panels* with field sizes of 6.3 cm × 6.3 cm and 12.6 cm × 12.6 cm were 42.0% ± 23.0% (ranging from 6.8% to 80.3) and 40.3% ± 9.8% (ranging from 17.0% to 63.1%), respectively. Those on the dose distributions of the *front panels* were 27.6% ± 12.9% (ranging from 10.0% to 70.4%) and 26.1% ± 13.3% (ranging from 7.9% to 52.9%), respectively. The calculated dose distributions on both panels were not coincident with the measured dose distributions.

#### Differences between the values of D_Rx_ from the calculated and measured dose distributions

**Fig 8:**
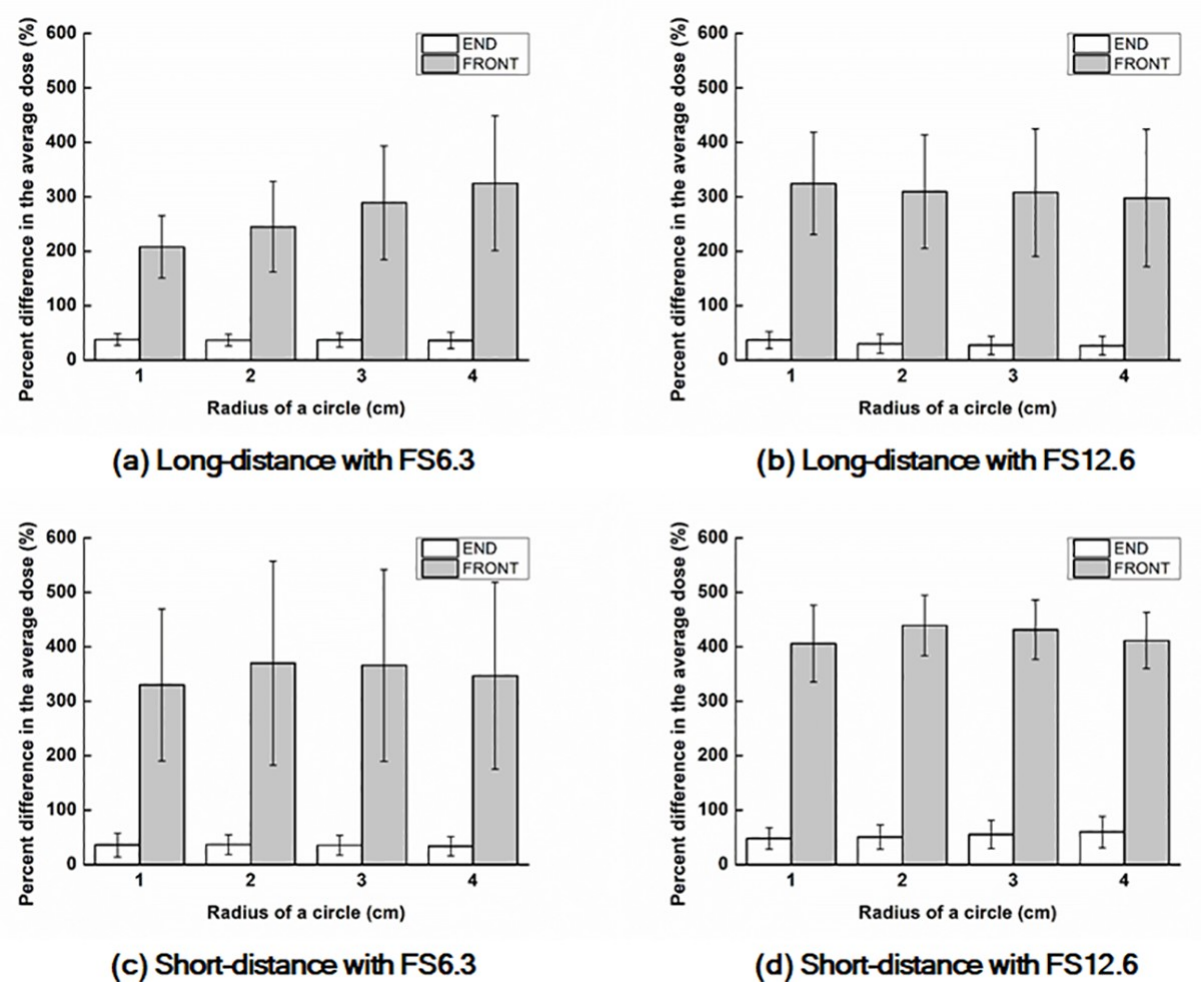
Average percent differences between the values of D_Rx_ from the calculated and measured dose distributions. The differences with a field size of 6.3 cm × 6.3 cm at 17 cm (a) and 10 cm (c) distances from CAX as well as those with a field size of 12.6 cm × 12.6 cm at 17 cm (b) and 10 cm (d) distances from CAX.

The average percent differences between the values of D_Rx_ from the calculated and measured dose distributions are plotted in Fig 8.

As shown in Fig 7, huge percent differences were observed at both the *end* and *front panels*. The percent differences between the calculations and measurements at the *front panels* were much larger than those at the *end panels*. This resulted from the smaller D_Rx_ values at the normalization points (maximum doses) of the *front panels* than those at the *end panels*. The average absolute differences in D_R1_ of the *front panels* with field sizes of 6.3 cm × 6.3 cm and 12.6 cm × 12.6 cm were 26.4 ± 13.0 cGy and 46.4 ± 17.5 cGy, respectively, while those of the *end panels* were 61.1 ± 23.0 cGy and 136.1 ± 41.7 cGy. Although the absolute differences at the *end panels* were larger than those at the *front panels*, the percent differences normalized to the maximum dose in the dose distributions of the *front panels* appeared to be larger than those of the *end panels* because the maximum doses at the *front panels* were smaller than those at the *end panels*. In general, the calculated doses were larger than those of the measured doses, *i*.*e*., doses calculated by the air-electron streams were generally overestimated compared to the measurements.

### Measured dose differences between the front and end panels

The average percent differences in the values of D_Rx_ from the measured dose distributions between the *front* and *end panels* are plotted in S1 Fig, which can be found in supporting information file. Doses at the *end panels* were always larger than those at the *front panels*.

### Measured dose differences between the large and small field sizes

The average percent differences in the values of D_Rx_ from the measured dose distributions between the field sizes of 12.6 cm × 12.6 cm and 6.3 cm × 6.3 cm are plotted in S2 Fig, which can be found in supporting information file. Doses with a large field size (12.6 cm × 12.6 cm) were always larger than those with a small field size (6.3 cm × 6.3 cm).

### Measured dose differences between the long and small distances from central axis

The average percent differences in D_Rx_ values from the measured dose distributions between the distances of 17 cm and 10 cm from CAX are plotted in S3 Fig, which can be found in supporting information file.

Doses at the long distance from CAX (17 cm) were always smaller than those at the short distance (10 cm).

### Correlations between the projected areas and doses on the panels

The calculated values of the projected areas of the beam cross-sections at the phantom surface on the panels are shown under various conditions of the present study in S1 Table, which can be found in supporting information file.

With an increase in the *phantom angles* and gantry angles, the projected areas increased. The projected areas on the *end panels* were larger than those on the *front panels*. The smallest and largest areas projected on the panels were 6.2 cm^2^ (gantry angle = 0°, *phantom angle* = 10°, field size = 6.3 cm × 6.3 cm, and on the *front panel*) and 109.4 cm^2^ (gantry angle = 30° and 330°, *phantom angle* = 30°, field size = 12.6 cm × 12.6 cm, and on the *end panel*), respectively.

**Fig 9:**
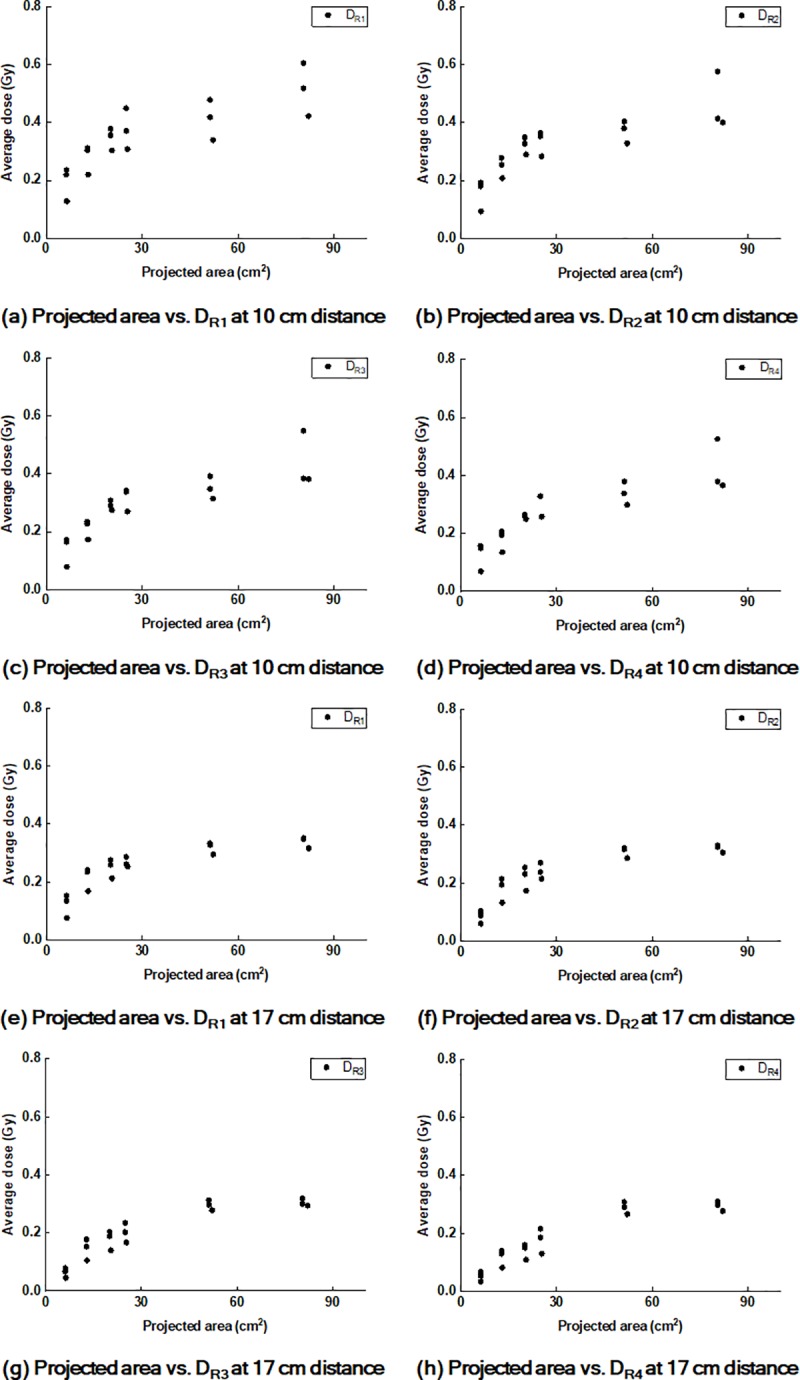
Plots of values of D_Rx_ according to the projected areas on the front panels. Plots of values of D_R1_ (a), D_R2_ (b), D_R3_ (c), and D_R4_ (d) on the front panels at the 10 cm distance from CAX according to the projected areas of the cross-section of the treatment beam at the phantom on the front panels. Plots of the values of D_R1_ (e), D_R2_ (f), D_R3_ (g), and D_R4_ (h) at the 17 cm distance from CAX according to the projected areas.

**Fig 10:**
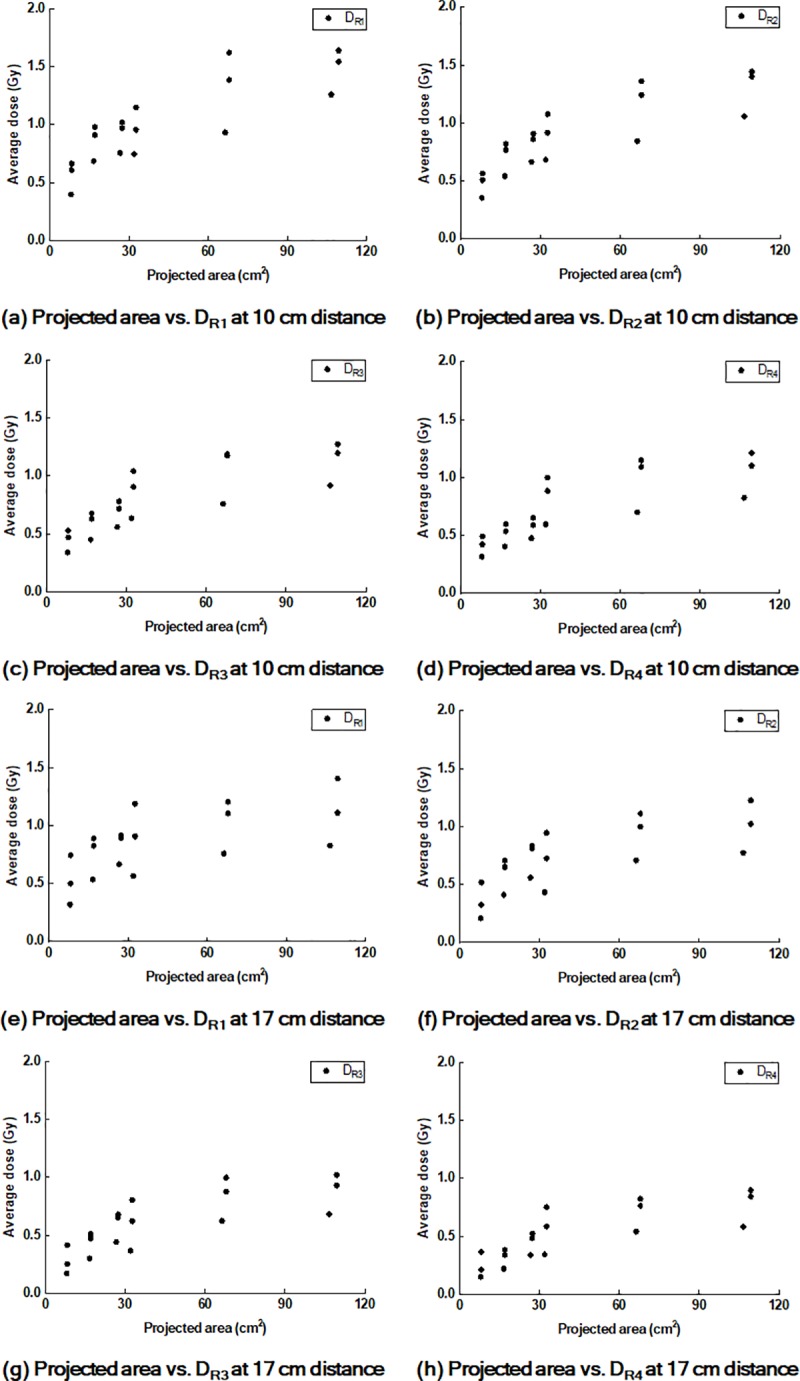
Plots of values of D_Rx_ according to the projected areas on the end panels. Plots of values of D_R1_ (a), D_R2_ (b), D_R3_ (c), and D_R4_ (d) on the end panels at 10 cm distance from CAX according to the projected areas of the cross-section of the treatment beam at the phantom on the end panels. Plots of the values of D_R1_ (e), D_R2_ (f), D_R3_ (g), and _DR_4 (h) at the 17 cm distance from CAX according to the projected areas.

The values of D_Rx_ according to the projected areas on the *front* and *end panels* are plotted in Figs 9 and 10, respectively.

The *r* values of the projected areas on the panels to the values of D_Rx_ are summarized in Table 9 with the corresponding *p* values.

**Table 9:** Spearman’s rank correlations between the projected areas on the panels and the values of D_Rx_.

Panel	D_R1_		D_R2_		D_R3_		D_R4_	
	*r*	*p*	*r*	*p*	*r*	*p*	*r*	*p*
Distance of 10 cm from CAX								
Front	0.811	< 0.001	0.891	< 0.001	0.901	< 0.001	0.892	< 0.001
End	0.876	< 0.001	0.922	< 0.001	0.938	< 0.001	0.919	< 0.001
Distance of 17 cm from CAX								
Front	0.898	< 0.001	0.890	< 0.001	0.876	< 0.001	0.875	< 0.001
End	0.757	< 0.001	0.851	< 0.001	0.885	< 0.001	0.910	< 0.001

*Abbreviations*: D_Rx_ = average dose in a circle drawn around the point of the maximum dose with a radius of *x* cm

## Discussion

In the present study, we investigated undesired irradiations outside of the treatment field by the air-electron stream during MR-IGRT for tumors located at superficial regions in the body when generating a slope between the patient’s body surface and the direction of the magnetic field in cases such as MR-IGRT for APBI. This phenomenon occurs owing to the same reason as the electron return effect during MR-IGRT, which is the Lorentz force [8, 9]. Although the air-electron streams were generated with the same reason as the electron return effect, its effect on the treatment is different from that of the electron return effect because the air-electron streams result in an undesired irradiation outside of the treatment field, while the electron return effect results in an increased dose deposition at the tissue−air interface [9]. Under various conditions, we investigated which factor increased the doses outside of the treatment field by the air-electron stream. We found that the doses by the air-electron streams increased with increasing *phantom angles*, increasing field sizes, and decreasing distances from the treatment field. We also found that the doses by the air-electron stream increased at gantry angles of 30° and 330° compared to those at a gantry angle of 0°. In addition, the doses on the *end panels* were always lager than those on the *front panels*. The conditions of the large *phantom angles*, large field sizes, measurement at the *end panels*, and oblique gantry angles result in an increase in the projected area of the treatment beam cross-section at the phantom on the panels. It is obvious that the increases in the *phantom angles* and field sizes, as well as oblique beams, to the phantom increase the projected area on the panels. The projected areas on the *end panels* are also larger than those on the *front panels* under an identical condition because the treatment beam diverges. Therefore, comprehensively reviewing the results, the increase in the projected area plays a major role in increasing the out-of-field doses by the air-electron streams. This is clearly identified in the correlations between the projected areas and the values of D_Rx_. We found very strong correlations, showing *r* values of up to 0.938, between the projected area and the values of D_Rx_ (all with *p* < 0.001). Accordingly, the largest dose in the present study was observed on the *end panel* with a field size of 12.6 cm × 12.6 cm at a gantry angle of 330° and *phantom angle* of 30°, which was 164.1 cGy (D_R1_).

In the case of exit beams, not only the increases in the projected areas owing to the beam divergence but also the vectors of the electrons scattered from the phantom to air might affect the increases in the doses outside of the treatment field by the air-electron stream. Since the electrons generated by the photon beams should escape the phantom to form the air-electron streams, the electrons scattered from the phantom at the beam exit would be more than those backscattered at the beam entrance considering the gamma ray direction into the phantom. This could increase doses on the *end panels* by the air-electron stream. However, the gamma rays at the exit were attenuated more than those at the entrance, which results in a decrease in the number of electrons scattered from the phantom. This could decrease doses on the *end panels* by the air-electron stream. Therefore, for the doses on the *end panels*, there were causes to both increase and decrease the doses on the *end panels*, simultaneously. Reviewing the results, the doses on the *end panels* were higher than those on the *front panels*. For further investigation, Monte Carlo simulation should be performed, and therefore, we will investigate the doses at the *end panels* with Monte Carlo simulation in the future.

The doses at a short distance from CAX were always larger than those at a large distance, which is irrelevant to the projected area sizes. This seems to be owing to the low energies of the scattered electrons in this study. The air-electron streams in the present study were generated from the Co-60 gamma rays (maximum energy of 1.33 MeV), and therefore, the electron energies should be lower than those of the maximum energy of the gamma ray [1]. With these low energies, most electrons in the air-electron streams might not propagate far away in air. Therefore, as increasing the distance from the treatment beam, doses by the air-electron stream decreased as shown in the results. If high-energy photon beams were used for MR-IGRT utilizing the MR-linac, such as MRIdian Linac (ViewRay Inc., Cleveland, OH), with a 6 MV photon beam or the Elekta MR-linac with a 7 MV photon beam (Elekta, Strockholm, Sweden), the air-electron streams could propagate farther than those presented in this study; therefore, it is necessary to be highly cautious for the air-electron streams when using MR-linac [15, 16].

Hackett *et al*. investigated the spiraling contaminant electrons utilizing the Elekta MR-linac, which increase surface doses outside of the treatment field. The spiraling contaminant electrons is quite similar to the air-electron stream in the present study since both are electrons with directional nature by the magnetic field and deposit doses outside of the treatment field. However, the spiraling contaminant electrons are generated in the components of the linac head, shielding, and in the air column though which the incident beam passes while the air-electron stream is generated by the secondary electrons scattered in air from a patient. In other words, the spiraling contaminant electrons are generated with the contaminant electrons while the air-electron streams are generated with the secondary electrons scattered in air from a patient. Therefore, the doses outside of the treatment field by the spiraling contaminant electrons were measured without a phantom in the study by Hackett *et al*. while the air-electron streams were measured with a phantom in the present study. The surface dose outside of the treatment field (field size = 10 cm × 10 cm) by the spiraling contaminant electrons was approximately 5% of the maximum dose at 5 cm distance from the field edge, which is much smaller than those of the present study although the photon beam energy of the Elekta MR-linac was larger than that of the ViewRay system (7 MV vs. 1.17 MeV and 1.33 MeV).

The calculated doses outside of the treatment field by the air-electron streams were not coincident with the measured ones. One of the reasons for this discrepancy might be the large dose calculation grid size of the MRIdian system in the present study, which was 3 mm. As mentioned above, to maintain the dose calculation accuracy based on the Monte Carlo algorithm, *i*.*e*., to reduce uncertainties in the calculated doses in the voxels due to small numbers of histories, as well as to enable fast dose calculation for the on-Table ART in the clinic, the dose calculation grid size in the present study was set as 3 mm following the manufacturer’s recommendation. However, the doses by the air-electron streams were surface doses, which should be assessed with a fine dose calculation resolution. The doses by the air-electron streams measured with the EBT3 films were doses at the depths of approximately 0.14 mm since the thickness of the EBT3 film is approximately 0.27 mm. Therefore, the doses on the panel surfaces calculated with a dose calculation grid size of 3 mm would be different from those measured with the films. Besides the dose calculation resolution, there might be other reasons for the discrepancy between the calculations and the measurements in this study. This will be investigated further in the future.

In an extreme case, the largest average dose inside a circle with a radius of 1 cm (area of approximately 3.14 cm^2^) by the air-electron stream at 10 cm distance from CAX was as large as 54.7% of the prescription dose and the area irradiated by equal to or larger than 50% of the prescription dose was 18.5 cm^2^. These are clinically significant undesired irradiations considering that the irradiated regions would be normal tissue far from the treatment volume [17]. However, these high dose irradiations outside of the treatment field would hardly occur during an actual treatment in the clinic since multiple beams are generally utilized for the actual treatment. In addition, because a patient’s body is generally thicker than the phantom used in this study, the exit dose would not be as high as those observed on the *end panels* in this study. In a previous study, we performed *in vivo* measurements of the doses by the air-electron streams during APBI, and found the average value of D_R1_ to be approximately 4% of the prescription dose, which is much smaller than those in the present study [7]. Owing to the low energies of the air-electron streams, we could easily shield these doses with only 1-cm-thick commercial build-up bolus (Superflab Bolus, Radiation Products Design Inc., Alvertville, MN). For the MR-linacs, materials thicker than 1-cm-thick bolus would be required to shield the air-electron streams owing to the higher energies of the photon beams than that of the Co-60 source.

## Conclusions

The undesired irradiation outside of the treatment field owing to the magnetic field when treating tumors located close to the patient’s surface is a unique feature of MR-IGRT, which requires careful caution. As shown in the results, the calculated doses by the air-electron streams are inaccurate compared to the measurements. We found that the doses by the air-electron streams increased with the projected area of the cross-section of the treatment beam on the irradiated surface. In this situation, shielding must be considered to prevent undesirable out-of-field irradiation. The undesired irradiation outside of the treatment field would be more problematic for MR-linac, which uses photons with higher energies than that of Co-60 source in the present study because the ranges in air as well as the penetrating powers of the air-electron streams would be larger than those presented in this study.

## Supporting information

S1 Fig Average percent differences in the values of D_Rx_ from the measured dose distributions between the front and end panels at the 17 cm (a) and 10 cm (b) distances from the central axis.
(DOCX)

S2 Fig Average percent differences in the values of D_Rx_ from the measured dose distributions between the field sizes of 12.6 cm × 12.6 cm and 6.3 cm × 6.3 cm plotted at 17 cm (a) and 10 cm (b) distances from the central axis.
(DOCX)

S3 Fig Plots of average percent differences in the values of D_Rx_ from the measured dose distributions between the distances of 17 cm and 10 cm from the central axis from the doses on the end panel (a) and front panel (b).
(DOCX)

S1 Table Areas of projection of the beam cross-section at the phantom surface on the panels. (DOCX)
