# Supplementary material for: Electron streams in air during magnetic-resonance image-guided radiation therapy
Source: PLoS One. 2019 May 15;14(5):e0216965. doi: 10.1371/journal.pone.0216965 (PMC6519819; doi:10.1371/journal.pone.0216965)
Supplement: S1 Fig — Average percent differences in the values of DRx from the measured dose distributions between the front and end panels at the 17 cm (a) and 10 cm (b) distances from the central axis. (DOCX) [file pone.0216965.s001.docx]

**Supporting information figure 1**


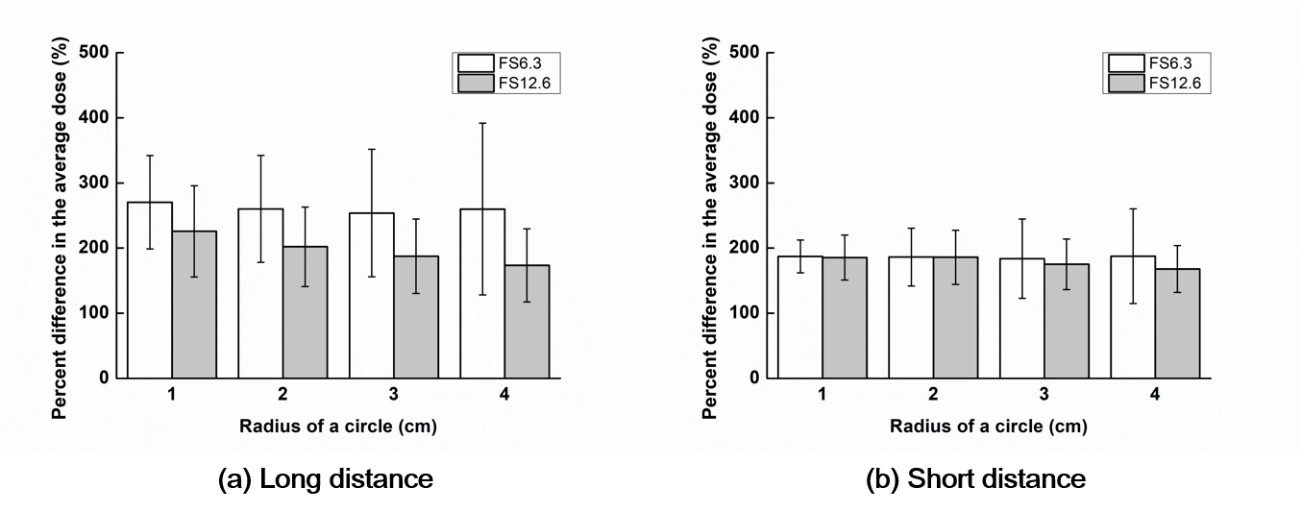


**S1 Fig.**

S1 Fig. Average percent differences in the values of D_Rx_ from the measured dose distributions between the front and end panels at the 17 cm (a) and 10 cm (b) distances from CAX.
