# Supplementary material for: Electron streams in air during magnetic-resonance image-guided radiation therapy
Source: PLoS One. 2019 May 15;14(5):e0216965. doi: 10.1371/journal.pone.0216965 (PMC6519819; doi:10.1371/journal.pone.0216965)
Supplement: S2 Fig — Average percent differences in the values of DRx from the measured dose distributions between the field sizes of 12.6 cm × 12.6 cm and 6.3 cm × 6.3 cm plotted at 17 cm (a) and 10 cm (b) distances from the central axis. (DOCX) [file pone.0216965.s002.docx]

**Supporting information figure 2**


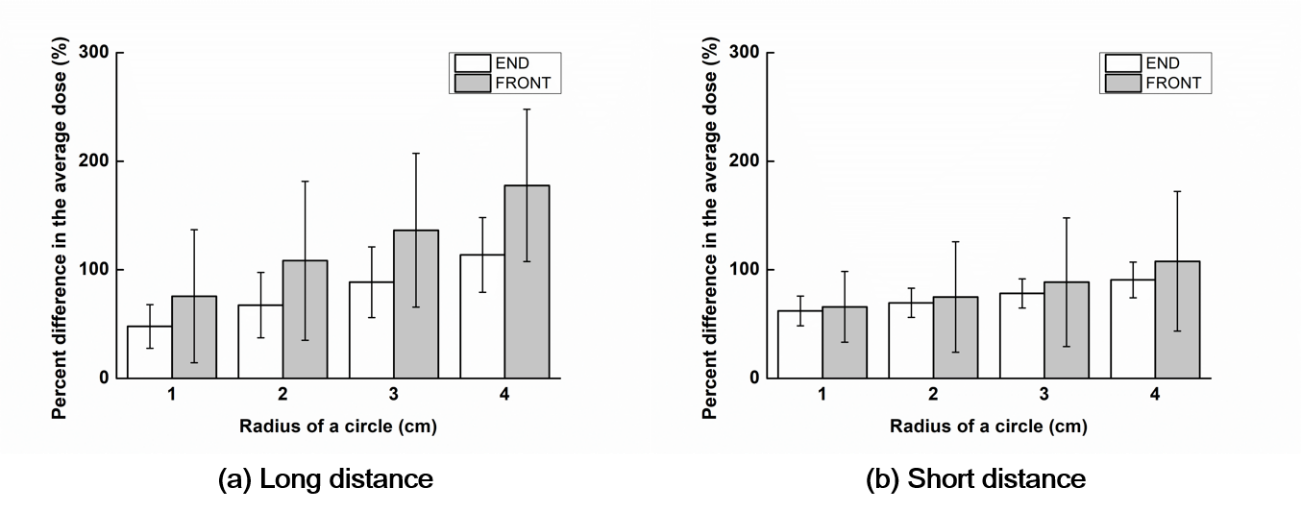


**S2 Fig.**

S2 Fig. Average percent differences in the values of D_Rx_ from the measured dose distributions between the field sizes of 12.6 cm × 12.6 cm and 6.3 cm × 6.3 cm plotted at 17 cm (a) and 10 cm (b) distances from CAX.
