# Supplementary material for: Electron streams in air during magnetic-resonance image-guided radiation therapy
Source: PLoS One. 2019 May 15;14(5):e0216965. doi: 10.1371/journal.pone.0216965 (PMC6519819; doi:10.1371/journal.pone.0216965)
Supplement: S3 Fig — Plots of average percent differences in the values of DRx from the measured dose distributions between the distances of 17 cm and 10 cm from the central axis from the doses on the end panel (a) and front panel (b). (DOCX) [file pone.0216965.s003.docx]

**Supporting information figure 3**


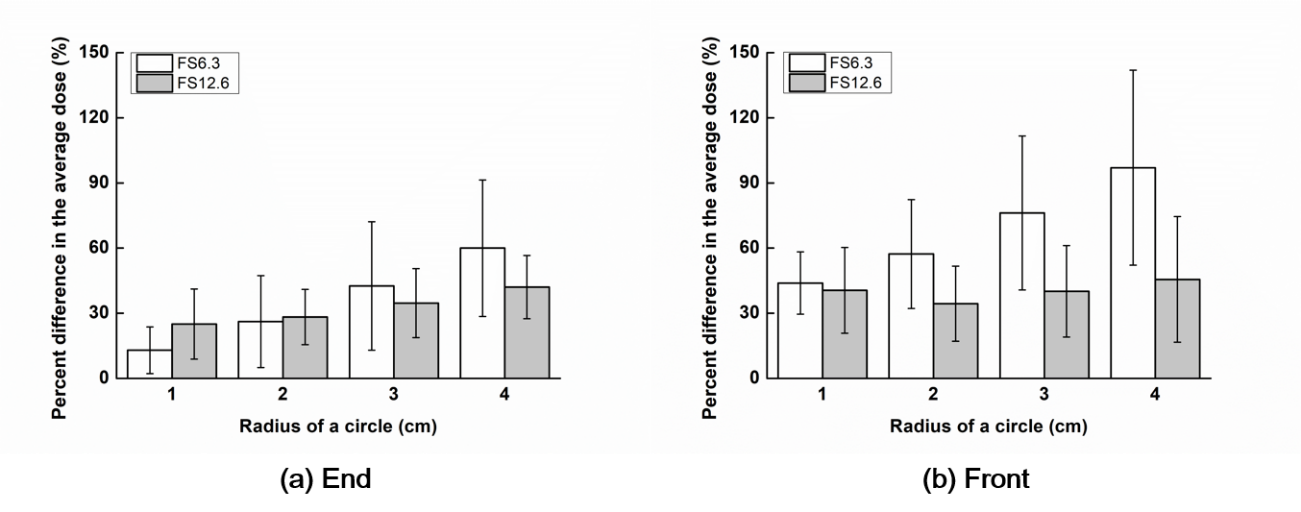


**S3 Fig.**

S3 Fig. Plots of average percent differences in the values of D_Rx_ from the measured dose distributions between the distances of 17 cm and 10 cm from CAX from the doses on the end panel (a) and front panel (b).
