# Supplementary material for: Electron streams in air during magnetic-resonance image-guided radiation therapy
Source: PLoS One. 2019 May 15;14(5):e0216965. doi: 10.1371/journal.pone.0216965 (PMC6519819; doi:10.1371/journal.pone.0216965)
Supplement: S1 Table — (DOCX) [file pone.0216965.s004.docx]

**Supporting information Table 1**

S1 Table. Areas of projection of the beam cross-section at the phantom surface on the panels

| Phantom angle | | 10° | | 20° | | 30° | |
| --- | --- | --- | --- | --- | --- | --- | --- |
| Gantry angle | | 0° | 30° (330°) | 0 | 30°  (330°) | 0 | 30° (330°) |
| Calculated values of the projected area (cm^2^) | | | | | | | |
| Projected on the  front panel | FS 6.3 cm 🞨 6.3 cm | 6.2 | 6.3 | 12.8 | 13.0 | 20.1 | 20.5 |
|  | FS 12.6 cm 🞨 12.6 cm | 24.9 | 25.4 | 51.2 | 52.1 | 80.5 | 82.1 |
| Projected on the  end panel | FS 6.3 cm 🞨 6.3 cm | 8.0 | 8.2 | 16.6 | 17.0 | 26.6 | 27.3 |
|  | FS 12.6 cm 🞨 12.6 cm | 31.9 | 32.6 | 66.3 | 67.9 | 106.6 | 109.4 |

*Abbreviations*: FS = field size
